# Pancreatic cancer surgery

**DOI:** 10.1186/s12893-023-02091-7

**Published:** 2023-07-07

**Authors:** Marcello Di Martino, Michael El Boghdady

**Affiliations:** 1 AORN, Cardarelli, Italy; 2 Guy’s and St Thomas’ NHS Foundation Trust, London, UK

This collection is intended to cover the latest advancements in the diagnosis and treatment of pancreatic cancer (PC). Pancreatic cancer is a deadly disease with an incidence that is rising at a rate of 0.5% to 1.0% per year and projected to become the second-leading cause of cancer death by 2030 [1]. It is often diagnosed at an advanced stage and despite advances in treatment, it presents a 5-year survival rate of around 10% [2]. Pancreatic ductal adenocarcinoma (PDAC) accounts for the majority (90%) of pancreatic neoplasms, and the other subtypes include periampullary and neuroendocrine tumours. The low survival rates of pancreatic cancer significantly affect the quality of life of patients, often with significant deterioration in terms of cognitive functions and mental changes [3].

Over the last few years, several research areas have been under intense scrutiny with the aim of improving clinical outcomes for patients with PC. Important progress has been made in understanding the genetic factors that contribute to pancreatic cancer. This knowledge can be used to develop more targeted treatments and screening methods. A promising development is the use of liquid biopsies for pancreatic cancer diagnosis and treatment. Liquid biopsies are non-invasive tests that can detect cancer cells RNA or DNA in a patient’s blood [4]. They could allow doctors to detect early-stage pancreatic cancer, which is often difficult to diagnose using traditional methods, monitor the progression of the disease and adjust treatment accordingly.

At the moment, surgical resection is the only curative option for patients with PC. Surgical procedures include pancreatoduodenectomy (PD), Pylorus preserving pancreaticoduodenectomy (PPPD) and distal pancreatectomy (DP).. They have been continuously refined to improve outcomes and reduce complications. Laparoscopic pancreatic surgery has been proven to improve patients’ outcomes and postoperative length of recovery [5]. In addition, advances in robotic surgery have been evolving in pancreatic surgery to overcome the limitations of standard laparoscopy regarding precise dissection and reconstruction. Similarly, the extent of resection as well as the reconstruction techniques are under a continuous scrutiny [6]. Despite that, post-operative clinically relevant pancreatic fistula (PO-CRPF) still remains the Achilles’ heel of this surgical procedure, but several mitigation strategies have been proposed to reduce the impact of post-operative complications on clinical outcomes [7].

Enhancements in perioperative systemic therapy have represented an important step forward in improving the outcomes of patients with pancreatic cancer. Several randomised controlled trials have demonstrated the benefits of perioperative chemotherapy in improving overall survival [8]. The CONKO-001 trial [9], comparing adjuvant chemotherapy with observation after surgical resection of PDAC, demonstrated that adjuvant chemotherapy should be standard of care. Newer regimens, such as FOLFIRINOX (5-fluorouracil, leucovorin, irinotecan, and oxaliplatin) or gemcitabine plus nab-paclitaxel, have shown even better results in recent clinical trials. The results of the APACT trial showed that adjuvant chemotherapy with gemcitabine plus nab-paclitaxel significantly improved disease-free survival compared to gemcitabine alone [8]. Similarly, the PRODIGE-24 CCTG PA6 RCT indicated that adjuvant treatment with modified FOLFIRINOX yields significantly longer survival than gemcitabine in patients with PDAC [10]. Whether neoadjuvant chemotherapy should be administered to resectable PC is still arguable. Regarding borderline, The PEOPANC and ESPAC-5 trial demonstrated that neoadjuvant therapy had the best survival compared with immediate surgery in patients with borderline resectable PDAC [11, 12].

Moreover, as anticipated above, recent advances in molecular profiling have highlighted the importance of identifying patient-specific molecular signatures to guide treatment decisions. Biomarkers such as microsatellite instability, homologous recombination deficiency and specific gene mutations such as BRCA may help guide the selection of patients for perioperative chemotherapy [13].

Finally, one of the most promising developments is the use of immunotherapy for pancreatic cancer. In a recent clinical trial, a combination of two immunotherapy drugs was shown to be effective in shrinking tumours in patients with advanced pancreatic cancer [14]. This is a significant breakthrough, as immunotherapy has not been effective in treating pancreatic cancer in the past.

In conclusion, this collection aims to offer a selection of articles that summarise the available evidence on the diagnosis and treatment of early and advanced PC (in the form of systematic review, ideally with meta-analysis), report on original experiences from centres with expertise and from the innovators of the field, provide the perspective of non-surgical staff, as well as patients and the public in relation to pancreatic cancer. The issue is open to both preclinical research (basic), translational research and clinical reports, and it will focus (but will not be limited to) the following topics:

- Risk factors for PC, pathways of PC development and progression;
- Strategies to reduce the incidence of PC, including screening tools;
- Preoperative assessment and optimisation to clinical outcomes;
- Surgical innovation and improvement of post-operative outcomes, including surgical management of resectable, borderline resectable and locally advanced PC;
- Newer treatment regimens in terms of adjuvant and neoadjuvant therapies, including personalised therapies and biological agents;
- Postoperative quality of life, patients and public experience in PC.

## Data Availability

Not applicable.
